# The Fly Pest

**Published:** 1921-05-14

**Authors:** 


### The Fly Pest.

Now is the time to begin to carry on a ruthless campaign against the fly pest. Every year about this time a few newspapers and periodical journals, at the risk of wearisome repetition, call attention to the danger to health from the common house-fly and to the need for systematic efforts on the part of the entire population to seek him in his lair and to prevent him from breeding. But usually the public takes no notice until it is too late, when the flies are present in their millions. Traps and fly-papers, then, are merely feeble expedients, and though a great deal of disease may be prevented by a scrupulous care in covering all forms of food and drink, it is almost impossible to make such safeguards absolutely proof against the revolting habits of the fly. There is absolutely nothing to be said in favour of the house-fly. It is fit only for extermination. It spreads typhoid fever, ophthalmia, and other serious diseases, is suspected of being the cause of some of which the proof is not quite conclusive, and is the deadly enemy of child-life in the late summer. Now is the time, we repeat, to attack the house-fly successfully, for now is the breeding-time. The way to prevent its breeding is to cover up all manure, refuse and decaying matter with unceasing care, and to ensure the complete destruction of all waste matter. Local authorities are as deeply concerned in this as individual householders. We agree with somebody who said the other day that it ought to be an actionable offence on the part of any local authority to resort to the disgusting practice of "tipping" its refuse in the open.

